# DNA methylation patterns provide insight into epigenetic regulation in the Pacific oyster (*Crassostrea gigas*)

**DOI:** 10.1186/1471-2164-11-483

**Published:** 2010-08-27

**Authors:** Mackenzie R Gavery, Steven B Roberts

**Affiliations:** 1 School of Aquatic and Fishery Sciences, University of Washington, 1122 NE Boat Street, Seattle, Washington, USA

## Abstract

**Background:**

DNA methylation is an epigenetic mechanism with important regulatory functions in animals. While the mechanism itself is evolutionarily ancient, the distribution and function of DNA methylation is diverse both within and among phylogenetic groups. Although DNA methylation has been well studied in mammals, there are limited data on invertebrates, particularly molluscs. Here we characterize the distribution and investigate potential functions of DNA methylation in the Pacific oyster (*Crassostrea gigas*).

**Results:**

Methylation sensitive PCR and bisulfite sequencing PCR approaches were used to identify CpG methylation in *C. gigas *genes and demonstrated that this species possesses intragenic methylation. *In silico *analysis of CpGo/e ratios in publicly available sequence data suggests that DNA methylation is a common feature of the *C. gigas *genome, and that specific functional categories of genes have significantly different levels of methylation.

**Conclusions:**

The Pacific oyster genome displays intragenic DNA methylation and contains genes necessary for DNA methylation in animals. Results of this investigation suggest that DNA methylation has regulatory functions in *Crassostrea gigas*, particularly in gene families that have inducible expression, including those involved in stress and environmental responses.

## Background

Epigenetic mechanisms induce changes in gene activity without alteration to the underlying DNA sequence [1]. Common epigenetic mechanisms include DNA methylation, histone modifications and non-coding RNA activity. The most well-studied of these is DNA methylation, which refers to the addition of a methyl group to position 5 of cytosines. In animals, this reaction is catalyzed by a family of enzymes called DNA (cytosine-5) methyltransferases (DNMTs) and occurs almost exclusively in CpG dinucleotides. DNA methylation is typically associated with transcriptional repression, and is primarily achieved by methylation in gene promoters [2-4]. The functional significance of DNA methylation in vertebrates includes providing genomic stability [5], regulation of imprinted genes [6] and X-chromosome inactivation [7]. In mammals, DNA methylation is essential for development and cell differentiation [8] and defects or unintended changes in DNA methylation can have deleterious consequences such as embryonic lethality [9] and tumorgenesis [10]. DNA methylation, like many epigenetic marks, may be heritable, therefore unintended changes as a result of environmental exposures or other processes can be passed on for multiple generations [11].

The extent of cytosine methylation varies considerably among eukaryotes. In vertebrates, approximately 70-80% of cytosines in CpG dinucleotides are methylated [12], a pattern referred to as global methylation. Invertebrates display a wide range of DNA methylation, from very limited methylation in *Drosophilia melanogaster *[13] and *Caenorhabditis elegans *[14] to a mosaic pattern of methylation in the sea urchin (*Strongylocentrotus purpuratus*) [15] and *Ciona intestinalis *[16,17]. Bird and Taggart [12] concluded that there were three general types of methylation patterns: the 'insect-type' which shows little to no methylation, the 'echinoderm-type', the genomes of which contain both methylated and non-methylated fractions, and the heavily methylated 'vertebrate-type'. Recent studies in the honey bee (*Apis mellifera*) indicate these patterns may be more complex [18,19]. In contrast to *D. melanogaster*, which lacks most of the classical DNMTs [20] and shows limited cytosine methylation [21], *A. mellifera *has a fully functional set of DNA methylation enzymes and shows substantial methylation across its genome [18].

In vertebrates, regulation of transcription by DNA methylation is accomplished by differential patterns of methylation in intergenic regions, namely gene promoters [2-4]. In contrast, there are no significant differences reported in the methylation status of gene promoters in invertebrates, where methylation appears to be targeted specifically to transcription units [17,22]. Computational analyses of the methylation status of *A. mellifera *genes have provided some of the first evidence supporting a regulatory role of intragenic DNA methylation in invertebrates [19,23]. In these studies, genes associated with general metabolic or 'housekeeping' functions were predicted to be hyper-methylated, whereas caste-specific genes were preferentially hypo-methylated. This functional clustering suggests DNA methylation serves to regulate gene transcription in *A. mellifera*, however, it is uncertain if this function is conserved across invertebrate taxa. Furthermore, it is unclear exactly how intragenic cytosine methylation directly affects transcription.

Studies in *A. mellifera *and others illustrate the diversity of DNA methylation patterns in invertebrate taxa and highlight gaps in our understanding of the evolutionary and functional significance of DNA methylation. One taxonomic group that has been notably absent from these investigations is the phylum Mollusca. Molluscs were first categorized as having 'echinoderm-type' DNA methylation patterns based on experimental evidence using the common mussel (*Mytilus edulis*) [12]. Since then, there has been little investigation of DNA methylation in molluscs with the exception of evidence suggesting the presence CpG methylation in the clam, *Donux truculus *[24]. In addition to increasing our understanding of the evolution of DNA methylation in invertebrate taxa, this study provides an opportunity to evaluate the Pacific oyster (*Crassostrea gigas*) as a model organism for analyzing DNA methylation in an aquatic species. Bivalve molluscs are important bioindicators [25] and elucidating the functional significance of DNA methylation in these organisms may prove valuable for understanding the effects of environmental stress in aquatic organisms. Here, we report the first investigation into DNA methylation profiles in the genome of the Pacific oyster. We confirm the presence of intragenic CpG methylation in *C. gigas*. We also demonstrate a relationship between predicted methylation status and gene function, suggesting that DNA methylation performs important regulatory functions in *C. gigas*. Implications of these findings are discussed in both an evolutionary and ecological context.

## Results

### Methylation Sensitive PCR

A Methylation Sensitive PCR (MSP) approach was used to identify specific methylated sites. Five genes associated with immune function were analyzed and methylation status determined (Table 1). Methylation status can be concluded based on the presence or absence of a PCR product in the methylation sensitive HpaII digest. Of the five genes analyzed, CpG methylation was confirmed for *heat shock protein 70 *(*hsp70*), whereas no methylation was detectable at restriction site(s) for the other sequences examined. The CpG observed to expected ratios (see Methods for calculation) are included in Table 1 for each gene. It should be noted that *hsp70 *has the lowest ratio of all the genes analyzed (0.57). This low ratio is predictive of a hyper-methylated status, which is confirmed here by MSP.

**Table 1:** Results of Methylation Specific PCR analysis for five *C. gigas *genes

Accession #	Best blast hit [Organism]	Undigested	HpaII	MspI	Number of restriction sites	CpG o/e
EW778441	heat shock protein 70 [*Crassostrea gigas*]	+	+	_	2	0.57

EW777519	heat shock protein 25 [*Danio rerio*]	+	_	_	3	0.81

EW778166	cytochrome P450 [*Haliotis diversicolor*]	+	_	_	1	0.85

EW778608	macrophage expressed protein 1-like protein [*Crassostrea gigas*]	+	_	_	6	1.08

EW778905	14-3-3 protein gamma (Protein kinase C inhibitor protein 1 [*Bos taurus*])	+	_	_	2	0.92

Results of methylation status of five genes associated with immune response by MSP. PCR was carried out on undigested, HpaII digested, and MspI digested DNA. Presence (+) or absence (-) of PCR product is indicated. Number of CCGG restriction sites in the indicated sequence and CpGo/e ratios are also provided.

### Bisulfite Sequencing PCR

**Figure 1:**
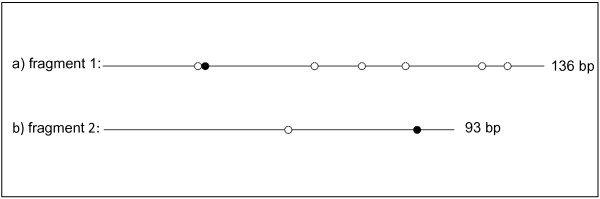
**Methylation status of two *C. gigas *DNA fragments by bisulfite sequencing**. Methylation status of a 136 bp (a) and 93 bp (b) fragment of *C. gigas *DNA as determined by bisulfite sequencing. Solid and open circles represent methylated and non-methylated CpG dinucleotides, respectively. One of four clones was determined to be methylated at the CpG indicated by the solid circle in (a) and 2 of 4 clones were determined to be methylated at the CpG dinucleotide indicated in (b).

In order to describe methylated cytosines outside of CCGG sites, Bisulfite Sequencing PCR (BSP) was used. Five genes predicted to be hyper-methylated, and five predicted to be hypo-methylated (based on CpG observed to expected ratio) were randomly selected for analysis. Valid PCR products were produced for two of the genes. This is a typical result as the conversion of unmethylated cytosines results in challenges for primer specificity. Four individual clones were sequenced for each of the two products. There was a 100% conversion rate for non-CpG cytosines for each of the clones sequenced. In the first fragment, a 136 bp fragment with homology to the amino terminal fragment of the human neuromedin-u receptor [Swiss-Prot: Q9GZQ4"], one of seven CpGs sites displayed methylation in 25% of the clones sequenced (Figure 1(a)). In a second fragment, one of two CpGs sites was determined to be methylated in 50% of the clones sequenced in a 93 bp region (Figure 1(b)). The latter sequence has significant homology to human bromodomain adjacent to zinc finger domain, 1A [Swiss-Prot: Q9NRL2].

### *In Silico *Analysis of *C. gigas *Transcriptome

The ratio of observed to expected CpG dinucleotides (CpGo/e) was used to predict methylation status in the *C. gigas *transcriptome. This approach is based on the known hyper-mutability of methylated cytosines, which readily deaminate to thymine residues [26]. This CpG mutation is not easily corrected by DNA repair machinery, and as a result consistently methylated regions of DNA are depleted of CpG dinucleotides over evolutionary time [27]. Consequently, regions of DNA with a low CpGo/e are predicted to be methylated, whereas regions with a high CpGo/e (approaching 1.0) are predicted to be unmethylated. This approach has been used to reliably predict methylation status across many taxonomic groups [17,19,22,28].

**Figure 2:**
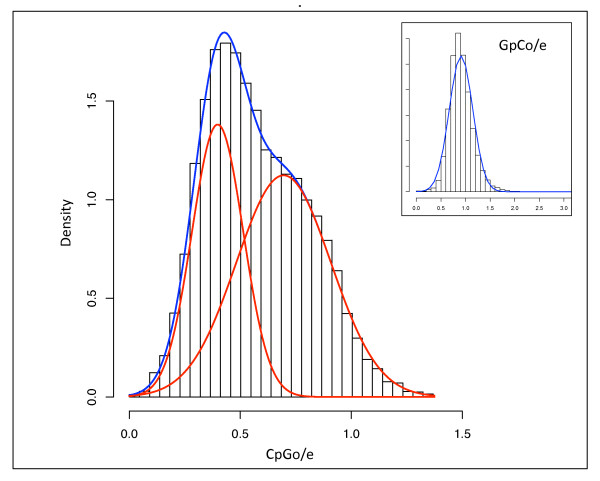
**Distribution of predicted methylation status of 12,210 annotated *C. gigas *transcripts measured computationally by CpGo/e ratio**. Probability density function of CpGo/e for 12,210 *C. gigas *contigs. Blue curve is fitted mixture model; red curves are scaled, normal mixture components with means of 0.40 and 0.70 respectively. For contrast, a control dinucleotide (GpCo/e) is also shown with the blue curve representing a normal, unimodal distribution (inset).

A non-redundant *C. gigas *contig database, 'GigasDatabase' version 6 [29] was utilized for this analysis. To ensure only CpG (and not GpC) dinucleotides were being evaluated, analysis was limited to annotated sequences. The probability density function of the CpGo/e for 12,210 annotated *C. gigas *expressed sequence tag (EST) contigs is illustrated in Figure 2. We find that the data fit a bimodal mixture model (blue curve) significantly better than a unimodal distribution. The red curves represent the scaled, normal mixture components, which have means of 0.40 (± 0.12 SD) and 0.70 (± 0.21 SD) respectively (Figure 2). A majority of the contigs have a CpGo/e less than 1.0.

The ratio of observed to expected GpC dinucleotide frequencies (GpCo/e) was calculated in order to be assured that the bimodal distribution of CpGo/e was not biased toward G+C content of specific genes as there are no known mechanisms for preferential depletion of the GpC dinucleotide. As predicted, the ratio of observed to expected GpC's approaches 1.0 following a unimodal Gaussian distribution (Figure 2 inset). In addition, there is a significant negative correlation between CpGo/e and TpGo/e (p = 0.00) indicating that the depletion of CpG dinucleotides is associated with the conversion of methylated CpG sites to TpG dinucleotides.

**Figure 3:**
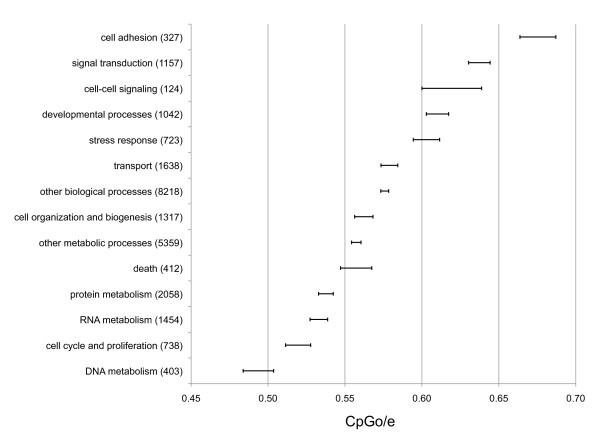
**Differential methylation between categories of genes involved in discrete biological processes as measured by CpGo/e**. Mean CpGo/e for 10,699 *C. gigas *contigs categorized by GO Slim term. Bars represent mean ± 1 standard error. The number of contigs in each category is listed in parenthesis.

In order to determine any functional difference that may exist among those genes with lower than expected CpGo/e ratios, data were analyzed in the context of each gene's biological process GO Slim term (Figure 3). Several biological processes have CpGo/e ratios that are significantly different from each other (see Additional file 1: Matrix of p-values for comparisons between GO Slim categories based on CpGo/e). Specifically, genes with lower CpGo/e ratios (predicted to be hyper-methylated) were associated with DNA metabolism, RNA metabolism, and cell cycle and proliferation. Biological processes with higher CpGo/e ratios (predicted to be hypo-methylated) include cell adhesion, cell-to-cell signalling and signal transduction. This analysis confirms that the normal mixture components described previously in Figure 2 are enriched with genes from particular functional categories.

## Discussion

Results of methylation specific PCR and bisulfite sequencing PCR indicate that the Pacific oyster (*Crassostrea gigas*) genome is methylated. Further evidence supporting the presence and importance of methylation in *C. gigas *is the identification of genes that encode DNA methyltransferases (DNMT), the family of proteins responsible for the enzymatic conversion of cytosine to 5-methylcytosine. Animals that lack DNA methylation such as *C. elegans *also lack essential DNMTs, while invertebrates with an intermediate level of DNA methylation such as honey bees, sea urchins and urochordates have the full set of DNMT genes [30]. Sequences with high homology to DNMT3 (responsible for *de novo *methylation), DNMT1 (associated with maintenance methylation), and methyl-CpG-binding domain protein 2 (mediation of the effects of DNA methylation) are present in a publicly available *C. gigas *contig database, GigasDatabase version 6 [29]. These annotated sequences can be found in GigasDatabase with accession numbers CU684371.p.cg.6 (e-value 1e-61), CU994437.p.cg.6 (e-value 2e-26), and AM861084.p.cg.6 (e-value 1e-11), respectively. While a DNMT2 homolog has not been identified, it may not be required for DNA methylation in *C. gigas *as it functions primarily as a tRNA methyltransferase and shows only weak DNA methyltransferase activity *in vitro *[31]. DNMTs are an evolutionarily conserved group of proteins, but show structural diversity both within and among taxa [32]. The evolutionary diversity of DNA methylation within and among phylogenetic groups provides justification for further evaluation of the functions of this epigenetic mark.

The presence of intragenic methylation in *C. gigas *is similar to that of other invertebrates that primarily exhibit intragenic DNA methylation patterns [33,17], the roles of which have been largely unexplored. Studies of DNA methylation in mammals have generally focused on promoter regions, where hyper-methylation of promoters inhibits initiation of transcription [2]. In contrast, invertebrate genomes do not show differentially methylated gene promoters [22]. One of the long-standing hypotheses is that intragenic DNA methylation prevents inappropriate initiation of transcription outside of promoter regions [34]; however new studies have begun to investigate a more active role for intragenic DNA methylation, namely in regulation of expression. For example, exonic DNA methylation has been shown to regulate transcription of the *phytochrome A *gene in *Arabidopsis thaliana *[35]. In humans, investigation of intragenic CpG islands (≥ 200 bp regions with G+C content of at least 50% and CpGo/e close to expected) has revealed that CpG islands in terminal exons may regulate transcription of non-coding RNAs [36]. Here, using BSP, we observed methylation variability in two CpG sites that may indicate cell-specific methylation. The function of intragenic DNA methylation in *C. gigas *cannot be conclusively determined from this study, but results of studies in other organisms suggest that it could be involved in either repression of transcription outside of transcription start sites and/or regulation of expression.

Within the transcriptome of the Pacific oyster, a significant difference in methylation pattern was observed across gene families. A majority of *C. gigas *genes analyzed were depleted in CpG dinucleotides (i.e. CpGo/e < 1.0) and show a significantly bimodal distribution, suggesting that DNA methylation is a common feature of the *C. gigas *transcriptome, and that certain groups of genes have significantly different levels of methylation. The bimodal distribution of CpGo/e is similar to the pattern observed in the honey bee *A. mellifera*, where authors reported a hyper-methylated fraction that was enriched in genes involved with general metabolic functions and a hypo-methylated fraction enriched with genes that are associated with caste-specific functions [19]. Similarly when *C. gigas *transcripts were clustered according to their functional annotations using GO Slim terms, we see that the two distributions are comprised of functionally distinct classes of genes with varying regulatory requirements. Specifically, genes predicted to be hyper-methylated are associated with housekeeping functions and those predicted to be hypo-methylated are associated with general immune functions. Hyper-methylation of intragenic regions of housekeeping genes is consistent between *C. gigas *and *A. mellifera *[19], but stands in contrast to observations in vertebrates, where distinct hypo-methylation of housekeeping gene promoters is associated with global expression [37]. Constitutive DNA methylation in housekeeping genes in *C. gigas *could be important for repressing transcription outside of promoter regions as previously discussed. It has also been proposed that hyper-methylation of housekeeping genes in *A. mellifera *indicates epigenetic control of gene activity in housekeeping genes [23]. Further experiments will be required to determine whether hyper-methylation of housekeeping genes plays a passive role in preventing inappropriate transcription or a more active role in maintaining expression in *C. gigas*.

Highest CpGo/e ratios were observed in genes involved in the oyster's innate immune response, including categories of cell adhesion, cell-cell signaling, and signal transduction. Our experimental data using MSP supports the predicted hypo-methylation of this class of genes as only 1 of the 5 immune related genes were methylated. Our results do not indicate that DNA methylation is entirely absent from genes in the hypo-methylated group as CpG depletion is still observed (CpGo/e 0.7) which stands in contrast to the hypo-methylated genes in *A. mellifera *(CpGo/e >1.0). One explanation as to why it would be advantageous for this class of genes to be hypo-methylated is that it allows for greater epigenetic flexibility and higher regulatory control. Oysters have been shown to have high phenotypic plasticity in response to environmental changes and stress [38,39] and it could be postulated that an epigenetic mark, such as DNA methylation, could provide this level of control. DNA methylation has been generally considered to be a less dynamic epigenetic mark, however, it has been reported in plants that DNA methylation levels are involved in regulating gene expression in response to stress and show active methylation and demethylation in response to various stressors [40-42]. It has been hypothesized from these studies that DNA methylation is a possible mechanism to impart protection against local stresses in future generations [43]. The identification of genes involved in demethylation in *C. gigas *would be an important step toward uncovering the nature of these epigenetic marks.

DNA methylation patterns have been shown to be heritable in mammalian taxa [44], and changes in DNA methylation patterns can persist for multiple generations [45]. Little work has been done to investigate heritability of DNA methylation in invertebrates, although a recent study of the crustacean, *Daphnia magna*, has shown transgenerational heritability of DNA methylation patterns after exposures to 5-azacytidine [46]. If DNA methylation does play a role in regulation of transcription in *C. gigas *it may provide a mechanism not only for regulating responses to stress, but also for adapting to local stressors through heritability of DNA methylation patterns. Investigating the potential of epigenetic control in mechanisms of local adaptation may prove useful in understanding impacts of anthropogenic inputs in aquatic ecosystems and populations. Likewise, it is possible that epigenetic mechanisms may provide an explanation for other phenomena associated with heritability such as inbreeding depression and hybrid vigour.

Elucidating functional significance of DNA methylation in aquatic invertebrates may change the way we study impacts of environmental change in aquatic organisms. A range of factors such as diet [47,48], xenobiotic chemicals [49], and endocrine disruptors [11] have been shown to disrupt DNA methylation patterns. These epigenetic disruptions are increasingly associated with disease susceptibility, which in some cases can be passed on for multiple generations [50]. Although these investigations have been performed almost exclusively in mammalian species, recent studies have reported a dose dependent relationship between concentration of mercury and cadmium and total DNA methylation in *D. magna *[46,51]. Understanding which environmental factors can affect DNA methylation and elucidating the functional significance of DNA methylation in these important bioindicator species will be major steps toward clarifying the complex interactions between the environment, gene expression, and organismal responses.

## Conclusions

The Pacific oyster genome displays methylation. *In silico *analysis reveals intragenic regions are targeted for methylation consistent with reports of methylation in other invertebrate species. Results of this investigation suggest that DNA methylation has regulatory functions in *Crassostrea gigas*, particularly in gene families involved in stress and environmental response. Experiments are underway in our lab to investigate relationships between the environment, DNA methylation, and control of gene expression to better characterize this process. In-depth analysis of methylation patterns in *Crassostrea gigas*, will help to advance the field of evolutionary epigenetics and will serve to illuminate functions of DNA methylation in invertebrates.

## Methods

### Animal collection & DNA isolation

Oysters used in this study were collected from naturalized *C. gigas *populations in Puget Sound, Washington. To isolate genomic DNA, 25 mg of gill tissue was processed according to the manufacturer's protocol using the DNeasy Blood & Tissue Kit (Qiagen, CA).

### Methylation Sensitive PCR

Oyster genomic DNA was enzyme digested with either HpaII or MspI. Five immune related genes containing one or more CCGG recognition sites and covering a broad range of predicted methylation status (based on CpGo/e) were selected from a set of ESTs generated from a cDNA library of plated hemocytes [52]. PCR primers were designed to flank one or more restriction sites. Primer sequences are provided in Additional file 2: Primer Sequences. Quantitative PCR was performed using digested (HpaII or MspI) and undigested samples using 1× Immomix Master Mix (Bioline USA Inc., Boston, MA), 2 uM SYTO-13 (Invitrogen, Carlsbad, CA) and 0.2 uM forward and reverse primers in an Opticon 2 System (Bio-Rad, Hercules, CA) with the following cycling conditions: 10 min at 95C, followed by 37 cycles of 15 sec at 95C, 30 sec at 55C, and 1 min at 72C and a final extension at 72C for 10 min. Results were scored qualitatively based on the presence or absence of amplification as determined by fluorescence.

### Bisulfite Conversion and Sequencing

Genomic DNA was bisulfite converted using the Epitect Bisulfite conversion kit (Qiagen, Carlsbad, CA). Briefly, 1.75 ug of DNA was subjected to treatment with sodium bisulfite at increased temperature to deaminate unmethylated cytosine residues to uracil. Following treatment, the solution was desulfonated on a column, washed and eluted.

To identify methylated cytosines in expressed regions of the oyster genome, Meth Primer [53] was used to design primers that flank multiple CpG sites, but do not contain CpGs. Primer sequences are provided in Additional file 2: Primer Sequences. The mean expected amplicon length for bisulfite primers was ~180 bp. PCR of bisulfite treated samples (54 ng/PCR reaction) was carried out using Taq DNA Polymerase Master Mix (Apex BioResearch Products, Research Triangle Park, NC) for 10 min at 95 C, followed by 40 cycles of 15 sec at 95 C, 30 sec at 55 C, and 1 min at 72C and a final extension at 72C for 10 min.

PCR products were separated using gel electrophoresis. Single bands were excised from the gel, purified using Ultra-DA purification columns (Ambion, Foster City, CA) and cloned using TOPO TA Cloning kit (Invitrogen). Four clones were sequenced for each primer pair. Methylation status was determined by comparing the sequence of bisulfite treated DNA to sequence of untreated DNA using Geneious 4.5.4 software (Biomatters Ltd., Aukland, NZ) and annotated using BLAST [54].

### *In Silico *Analysis: Predicted DNA Methylation Status

For *in silico *analysis, the non-redundant *C. gigas *expressed sequence tag (EST) contig database, 'GigasDatabase' version 6 (http://public-contigbrowser.sigenae.org:9090/Crassostrea_gigas/index.html, [29]), was utilized. Analysis was limited to annotated sequences (n = 12,210) in order to be confident that sequences were reported in the 5' to 3'direction. It should be noted that this transcriptomic dataset is appropriate for predicting methylation status of the *C. gigas *genome as investigation into other invertebrate species shows that DNA methylation is specifically targeted to transcribed regions of the genome [17,12].

CpG observed/expected ratio (CpGo/e) was calculated using the following equation where *l *is the number of nucleotides in the contig:

$$\text{CpGo/e}=\frac{\text{number}\;\text{of}\;\text{CpG}}{\text{number}\;\text{of}\;\text{C}\times \text{number}\;\text{of}\;\text{G}}\times \frac{l^{2}}{l-1}$$

To evaluate the distribution of Pacific oyster contigs, a mixture model was fit to the CpGo/e ratios using the mixtools package [55] in R [56] yielding a two component mixture where *p*_1 _+ *p*_2 _= 1. Hence the data C_*i*_, are distributed as:

$${\text{C}}_{\text{i}}\sim {\text{p}}_{1}\text{N}(\mu_{1},\sigma_{1})+{\text{p}}_{2}\text{N}(\mu_{2}\sigma_{2}).$$

The log likelihood statistic of the bimodal mixture model was compared to the normal null model to test for a significant improvement in fit.

In order to evaluate the variation of CpGo/e within and among functional classes of genes, contigs from the GigasDatabase annotated with a biological process GO term (n = 10,699 contigs) were assigned a functional group based on the MGI GO Slim database http://www.informatics.jax.org[57]. Since each contig may have multiple GO terms, contigs were allowed to fall into multiple GO Slim bins. However, to avoid weighting within a single category, an individual contig was not allowed to be included more than once in a single GO Slim bin. The mean CpGo/e and standard errors were calculated for each GO Slim term. A one-way ANOVA followed by Tukey's test for multiple comparisons was performed using SPSS software (SPSS Inc., Chicago, IL). A significance level of p < 0.05 was accepted.

## Authors' contributions

MG and SR conceived of the study and prepared the manuscript. MG carried out the laboratory procedures. All authors read and approved the final manuscript.

## Supplementary Material

Additional file 1 **Matrix of p-values for comparisons between GO Slim categories based on CpGo/e**. CpGo/e for GO Slim categories were compared with Tukey's multiple comparison test. This file contains the p-values of each comparison. Significant differences (p < 0.05) are highlighted. Click here for file

Additional file 2 **Primer Sequences**. This file contains primer sequences used for methylation sensitive PCR and bisulfite sequencing PCR analysis. Click here for file

## References

[B1] Jablonka E Lamb M The Changing Concept of Epigenetics Ann N Y Acad Sci 2002 981 82 96 10.1111/j.1749-6632.2002.tb04913.x 12547675

[B2] Boyes J Bird A Repression of genes by DNA methylation depends on CpG density and promoter strength: Evidence for involvement of a methyl-CpG binding protein EMBO J 1992 11 327 333 1310933 10.1002/j.1460-2075.1992.tb05055.x PMC556453

[B3] Kass SU Pruss D Wolffe AP How does DNA methylation repress transcription? Trends Genet 1997 13 444 449 10.1016/S0168-9525(97)01268-7 9385841

[B4] Hsieh CL Dependence of transcriptional repression on CpG methylation density Mol Cell Biol 1994 14 5487 5494 7518564 10.1128/mcb.14.8.5487-5494.1994 PMC359068

[B5] Maloisel L Rossignol JL Suppression of crossing-over by DNA methylation in *Ascobolus* Genes Dev 1998 12 1381 1389 10.1101/gad.12.9.1381 9573054 PMC316785

[B6] Bell AC Felsenfeld G Methylation of a CTCF-dependent boundary controls imprinted expression of the Igf2 gene Nature 2000 405 482 5 10.1038/35013100 10839546

[B7] Csankovszki G Nagy A Jaenisch R Synergism of Xist Rna, DNA Methylation, and Histone Hypoacetylation in Maintaining X Chromosome Inactivation J Cell Biol 2001 153 773 784 10.1083/jcb.153.4.773 11352938 PMC2192370

[B8] Okano M Bell DW Haber DA Li E DNA methyltransferases Dnmt3a and Dnmt3b are essential for de novo methylation and mammalian development Cell 1999 99 247 257 10.1016/S0092-8674(00)81656-6 10555141

[B9] Li E Bestor TH Jaenisch R Targeted mutation of the DNA methyltransferase gene results in embryonic lethality Cell 1992 69 915 926 10.1016/0092-8674(92)90611-F 1606615

[B10] Jones PA Baylin SB The epigenomics of cancer Cell 2007 128 683 92 10.1016/j.cell.2007.01.029 17320506 PMC3894624

[B11] Anway MD Skinner MK Epigenetic Transgenerational Actions of Endocrine Disruptors Endocrinology 2006 147 43 49 10.1210/en.2005-1058 16690803

[B12] Bird AP Taggart MH Variable patterns of total DNA and rDNA methylation in animals Nucleic Acid Res 1980 8 1485 1497 10.1093/nar/8.7.1485 6253937 PMC324011

[B13] Gowher H Leismann O Jeltsch A *DNA of Drosophila melanogaster contains 5-methylcytosine* EMBO J 2000 19 6918 6923 10.1093/emboj/19.24.6918 11118227 PMC305887

[B14] Simpson VJ Johnson TE Hammen RF *Caenorhabditis elegans *DNA does not contain 5-methylcytosine at any time during development or aging Nucleic Acids Res 1986 14 6711 6719 10.1093/nar/14.16.6711 3748820 PMC311675

[B15] Bird AP Taggart MH Smith BA Methylated and unmethylated DNA compartments in the sea urchin genome Cell 1979 17 889 901 10.1016/0092-8674(79)90329-5 487434

[B16] Simmen MW Bird AP Sequence analysis of transposable elements in the sea squirt, *Ciona intestinalis* Mol Biol Evol 2000 17 1685 1693 11070056 10.1093/oxfordjournals.molbev.a026267

[B17] Suzuki MM Kerr ARW De Sousa D Bird A CpG methylation is targeted to transcription units in an invertebrate genome Genome Res 2007 17 625 631 10.1101/gr.6163007 17420183 PMC1855171

[B18] Wang Y Jorda M Jones PL Maleszka R Ling X Robertson HM Mizzen CA Peinado MA Robinson GE Functional CpG methylation system in a social insect Science 2006 314 645 647 10.1126/science.1135213 17068262

[B19] Elango N Hunt BG Goodisman MAD Yi S DNA methylation is widespread and associated with differential gene expression in castes of the honeybee, *Apis mellifera* Proc Natl Acad Sci USA 2009 106 11206 11211 10.1073/pnas.0900301106 19556545 PMC2708677

[B20] Hung MS Karthikeyan N Huang B Koo HC Kiger J Shen CJ Drosophila proteins related to vertebrate DNA (5-cytosine) methyltransferases Proc Natl Acad Sci USA 1999 96 11940 11945 10.1073/pnas.96.21.11940 10518555 PMC18391

[B21] Lyko F Ramsahoye BH Jaenisch R DNA methylation in Drosophila melanogaster Nature 2000 408 538 540 10.1038/35046205 11117732

[B22] Elango N Yi SV DNA Methylation and Structural and Functional Bimodality of Vertebrate Promoters Mol Biol and Evol 2008 25 1602 1608 10.1093/molbev/msn110 18469331

[B23] Foret S Kucharski R Pittelkow Y Lockett GA Maleszka R Epigenetic regulation of the honey bee transcriptome: unravelling the nature of methylated genes BMC Genomics 2009 10 472 10.1186/1471-2164-10-472 19828049 PMC2768749

[B24] Petrović V Pérez-García C Pasantes JJ Šatović E Prats E Plohl M A GC-rich satellite DNA and karyology of the bivalve mollusk *Donax trunculus: *a dominance of GC-rich heterochromatin Cytogenet Genome Res 2009 124 63 71 10.1159/000200089 19372670

[B25] Markert BA Breure AM Zechmeister HG Markert BA, Breure AM, Zechmeister HG Molluscs as Bioindicators Bioindicators and Biomonitors 2003 Amsterdam: Elsevier Science 577 634

[B26] Coulondre C Miller JH Farabaugh PJ Gilbert W Molecular basis of base substitution hotspots in *Escherichia coli* Nature 1978 274 775 780 10.1038/274775a0 355893

[B27] Schorderet DF Gartler SM Analysis of CpG suppression in methylated and nonmethylated species Proc Natl Acad Sci USA 1992 89 957 961 10.1073/pnas.89.3.957 1736311 PMC48364

[B28] Shimizu TS Takahashi K Tomita M CpG distribution patterns in methylated and non-methylated species Gene 1997 205 103 107 10.1016/S0378-1119(97)00542-8 9461383

[B29] Fleury E Huvet A Lelong C de Lorgeril J Boulo V Gueguen Y Bachère E Tanguy A Moraga D Fabioux C Lindeque P Shaw J Reinhardt R Prunet R Davey G Lapègue S Sauvage C Corporeau C Moal J Gavory F Wincker P Moreews F Klopp C Mathieu M Boudry P Favrel B Generation and analysis of a 29,745 unique Expressed Sequence Tags from the Pacific oyster (Crassostrea gigas) assembled into a publicly accessible database: the GigasDatabase BMC Genomics 2009 10 341 10.1186/1471-2164-10-341 19640306 PMC2907693

[B30] Cañestro C Yokoi H Postlethwait JH Evolutionary developmental biology and genomics Nat Rev Genet 2007 8 932 942 10.1038/nrg2226 18007650

[B31] Schaefer M Lyko F Solving the Dnmt2 enigma Chromosoma 2010 119 35 40 10.1007/s00412-009-0240-6 19730874

[B32] Colot V Rossignol J Eukaryotic DNA methylation as an evolutionary device BioEssays 1999 21 402 411 10.1002/(SICI)1521-1878(199905)21:5<402::AID-BIES7>3.0.CO;2-B 10376011

[B33] Tweedie S Charlton J Clark V Bird A Methylation of genomes and genes at the invertebrate-vertebrate boundary Mol Cell Biol 1997 17 1469 1475 9032274 10.1128/mcb.17.3.1469 PMC231872

[B34] Bird AP Gene number, noise reduction and biological complexity Trends Genet 1995 11 94 100 10.1016/S0168-9525(00)89009-5 7732579

[B35] Chawla R Nicholson SJ Folta KM Srivastav V Transgene-induced silencing of *Arabidopsis *phytochrome A gene via exonic methylation Plant J 2007 52 1105 1118 10.1111/j.1365-313X.2007.03301.x 17931351

[B36] Medvedeva Y Fridman MV Oparina NJ Malko DB Ermakova EO Kulakovskiy IV Heinzel A Makeev V Intergenic, gene terminal, and intragenic CpG islands in the human genome BMC Genomics 2010 11 48 10.1186/1471-2164-11-48 20085634 PMC2817693

[B37] Weber M Schubeler D Genomic patterns of DNA methylation: targets and function of an epigenetic mark Current Opin Cell Biol 2007 19 273 280 10.1016/j.ceb.2007.04.011 17466503

[B38] Hamdoun AM Cheney DP Cherr GN Phenotypic Plasticity of HSP70 and HSP70 Gene Expression in the Pacific Oyster (*Crassostrea gigas*): Implications for Thermal Limits and Induction of Thermal Tolerance Biol Bull 2003 205 160 169 10.2307/1543236 14583513

[B39] Honkoop PJC Bayne BL Drent J Flexibility of size of gills and palps in the Sydney rock oyster *Saccostrea glomerata *(Gould, 1850) and the Pacific oyster *Crassostrea gigas *(Thunberg, 1793) J Exp Mar Biol Ecol 2003 282 113 133 10.1016/S0022-0981(02)00463-X

[B40] Kovarik A Koukalova B Bezdek M Opatrn Z Hypermethylation of tobacco heterochromatic loci in response to osmotic stress Theor Appl Genet 1997 95 301 306 10.1007/s001220050563

[B41] Steward N Ito M Yamaguchi Y Koizumi N Sano H Periodic DNA Methylation in Maize Nucleosomes and Demethylation by Environmental Stress J Biol Chem 2002 277 37741 37746 10.1074/jbc.M204050200 12124387

[B42] Choi CS Sano H Abiotic-stress induces demethylation and transcriptional activation of a gene encoding a glycerophosphodiesterase-like protein in tobacco plants Mol Genet Genome 2007 277 589 600 10.1007/s00438-007-0209-1 17273870

[B43] Chinnusamy V Zhu J Epigenetic regulation of stress responses in plants Current Opin Plant Biol 2009 12 133 139 10.1016/j.pbi.2008.12.006 PMC3139470 19179104

[B44] Anway MD Cupp AS Uzumcu M Skinner MK Epigenetic transgenerational actions of endocrine disruptors and male fertility Science 2005 308 1466 1469 10.1126/science.1108190 15933200 PMC11423801

[B45] Cropley JE Suter CM Beckman KB Martin DIK Germ-line epigenetic modification of the murine A(vy) allele by nutritional supplementation Proc Natl Acad Sci USA 2006 103 17308 1731 10.1073/pnas.0607090103 17101998 PMC1838538

[B46] Vandegehuchte MB Lemière F Vanhaecke L Berghe VW Janssen CR Direct and transgenerational impact on Daphnia magna of chemicals with a known effect on DNA methylation Comp Biochem Physiol C Toxicol Pharmacol in press 10.1016/j.cbpc.2009.11.007 19961956

[B47] Wilson MJ Shivapurkar N Poirier LA Hypomethylation of hepatic nuclear DNA in rats fed with a carcinogenic methyl-deficient diet Biochem J 1984 218 987 990 6721844 10.1042/bj2180987 PMC1153433

[B48] Dolinoy DC Weidman JR Waterland RA Jirtle RL Maternal Genistein Alters Coat Color and Protects *A^vy ^*Mouse Offspring from Obesity by Modifying the Fetal Epigenome Environ Health Perspect 2006 114 567 572 10.1289/ehp.8700 16581547 PMC1440782

[B49] Sutherland JE Costa M Epigenetics and the environment Ann NY Acad Sci 2003 983 151 160 10.1111/j.1749-6632.2003.tb05970.x 12724220

[B50] Jirtle RL Skinner MK Environmental epigenomics and disease susceptibility Nat Rev Genet 2007 8 253 262 10.1038/nrg2045 17363974 PMC5940010

[B51] Vandegehuchte MB Vanholme B Haegeman A Gheysen G Janssen CR Occurrence of DNA methylation in Daphnia magna and influence of multigeneration Cd exposure Environ Int 2009 35 700 706 10.1016/j.envint.2009.01.002 19249097

[B52] Roberts S Goetz G White S Goetz F Analysis of genes isolated from plated hemocytes of the Pacific oyster, *Crassostrea gigas* Mar Biotechnol 2009 11 24 44 10.1007/s10126-008-9117-6 18622569

[B53] Li LC Dahiya R MethPrimer: designing primers for methylation PCRs Bioinformatics 2002 18 1427 31 10.1093/bioinformatics/18.11.1427 12424112

[B54] Altschul SF Gish W Miller W Myers EW Lipman DJ Basic local alignment search tool J Mol Biol 1990 215 403 410 2231712 10.1016/S0022-2836(05)80360-2

[B55] Young DS Hunter DR Elmore RT Xuan F Hettmansperger TP Thomas H The mixtools Package Version 0.2.0: Tools for Mixture Models R Package Version 0.2.0 2007 http://www.r-project.org

[B56] R Development Core Team R: A language and environment for statistical computing http://www.R-project.org

[B57] Mouse Genome Database (MGD) at the Mouse Genome Informatics http://www.informatics.jax.org

