# Supplementary material for: DNA methylation patterns provide insight into epigenetic regulation in the Pacific oyster (Crassostrea gigas)
Source: BMC Genomics. 2010 Aug 27;11:483. doi: 10.1186/1471-2164-11-483 (PMC2996979; doi:10.1186/1471-2164-11-483)
Supplement: Additional file 1 — Matrix of p-values for comparisons between GO Slim categories based on CpGo/e. CpGo/e for GO Slim categories were compared with Tukey's multiple comparison test. This file contains the p-values of each comparison. Significant differences (p < 0.05) are highlighted. [file 1471-2164-11-483-S1.PDF]

Additional File 1. Matrix of p-values for comparisons between GO Slim categories based on CpGo/e

	cell adhesion	signal transduction	cell-cell signaling	developmental processes	stress response	transport	other biological processes	cell organization and biogenesis	other metabolic processes	death	protein metabolism	RNA metabolism	cell cycle and proliferation	DNA metabolism
cell adhesion		4.11E-2	5.13E-1	1.36E-5	2.81E-6	6.89E-12	6.43E-12	6.43E-12	6.43E-12	7.79E-12	6.43E-12	6.43E-12	6.43E-12	6.43E-12
signal transduction			1.00	0.32E-1	6.48E-2	1.15E-9	6.43E-12	6.43E-12	6.43E-12	5.18E-8	6.43E-12	6.43E-12	6.43E-12	6.43E-12
cell-cell signaling				1.00	9.94E-1	4.63E-1	3.15E-1	7.56E-2	2.79E-2	9.55E-2	7.55E-4	3.82E-4	4.23E-5	2.56E-7
developmental processes					1.00	2.49E-2	2.32E-4	1.34E-5	2.71E-10	3.74E-3	6.43E-12	6.43E-12	6.43E-12	6.43E-12
stress response						5.12E-1	1.21E-1	6.62E-3	3.76E-5	6.83E-2	1.79E-9	1.08E-9	1.89E-10	6.74E12
transport							1.00	7.24E-1	4.73E-2	9.04E-1	2.12E-6	1.46E-6	2.73E-7	4.38E-10
other biological processes								6.74E-1	2.50E-4	9.38E-1	2.96E-10	1.98E-9	7.19E-9	3.72E-11
cell organization and biogenesis									1.00	1.00	1.14E-1	5.22E-2	3.94E-3	5.65E-6
other metabolic processes										1.00	3.55E-2	1.76E-2	1.58E-3	1.92E-6
death											9.31E-1	8.07E-1	2.65E-1	2.96E-3
protein metabolism												1.00	8.67E-1	1.76E-2
RNA metabolism													9.90E-1	7.62E-2
cell cycle and proliferation														7.91E-1
DNA metabolism														
