# Supplementary material for: DNA methylation patterns provide insight into epigenetic regulation in the Pacific oyster (Crassostrea gigas)
Source: BMC Genomics. 2010 Aug 27;11:483. doi: 10.1186/1471-2164-11-483 (PMC2996979; doi:10.1186/1471-2164-11-483)
Supplement: Additional file 2 — Primer Sequences. This file contains primer sequences used for methylation sensitive PCR and bisulfite sequencing PCR analysis. [file 1471-2164-11-483-S2.PDF]

Additional Table 2. Primer sequences

Methylation Sensitive PCR:

Accession # [NCBI]	Best BLAST hit [Organism]	5' primer sequence	3'primer sequence
EW778441	heat shock protein 70 [*C. gigas*]	AGGGTATCGATTTCTACACAAG	GTTTCTCTTGATGAGATTGGTC
EW777519	heat shock protein 25 [*Danio rerio*]	AAATGAGCAAAATATTTAACGAGGA	TGGGATGGTAAGGATCAAGG
EW778166	cytochrome P450 [*Haliotis diversicolor*]	ATATTGGAGCCCTCGTTGTG	TGAGCGCAGAGAAACTTCAA
EW778608	macrophage expressed protein 1-like protein [*C. gigas*]	CGGAACCGAAGTAGATGGAA	ACGCTCATAAACGAGGCACT
EW778905	14-3-3 protein gamma (Protein kinase C inhibitor protein 1) [*Bos taurus*]	ATAGAGCGGAAGCCGTGATA	TAACTCGCAAGCAGTGTTGG

Bisulfite Sequencing PCR:

Accession # [GigasDatabase[1]]	Best BLAST hit [Organism]	5' primer sequence	3'primer sequence
AM858698.p.cg.6	neuromedin-u receptor [*H. sapiens*]	AGTTTTATATTGATTTTTGGAGAG	AAATTCTTCTCAAAATACATTCTTC
AM860932.p.cg.6	bromodomain adj. to zinc finger protein [*H. sapiens*]	TTGTAAATTGATAAATGAAATATTT	AAAATTCTTAAAAACCTTCTCCTC

[1] http://public-contigbrowser.sigenae.org:9090/Crassostrea_gigas/index.html
